# Super-refractory status epilepticus in a woman with *Aeromonas caviae* meningitis: a rare case report and review of the literature

**DOI:** 10.3389/fmed.2024.1410762

**Published:** 2024-07-01

**Authors:** Yanlang He, Jia Liu, Sheng Wei, Jianyong Chen

**Affiliations:** ^1^Medical College of Nanchang University, Nanchang, China; ^2^Jiangxi Provincial People's Hospital, The First Affiliated Hospital of Nanchang Medical College, Nanchang, China; ^3^Department of Geriatrics, Shaoyang Central Hospital, Shaoyang, China

**Keywords:** meningitis, *Aeromonas caviae*, super-refractory status epilepticus, multi-drug combination, anesthetic drug, non-anesthetic anti-epileptic drugs

## Abstract

Currently, there is a lack of knowledge regarding *Aeromonas caviae* meningitis. We report the first case of super-refractory status epilepticus (SRSE) in a woman with *Aeromonas caviae* meningitis. The case report demonstrates that this condition can lead to severe SRSE. Effective treatment for epilepsy is crucial for improving the prognosis for similar patients. According to Gomes et al.'s consensus protocol for SRSE, using a combination of up to one anesthetic drug and three non-anesthetic anti-epileptic drugs may be helpful and important in managing SRSE that is caused by *Aeromonas caviae* meningitis.

## Introduction

The *Aeromonas* species are a facultatively anaerobic, gram-negative, short rod-shaped bacteria that induce gastrointestinal and extra-intestinal infections such as soft tissue infection, osteomyelitis, endocarditis, and meningitis (1, 2). Previously, a case of *Aeromonas caviae* shunt infection and meningitis was reported in a newborn girl (3). However, there are no reported cases of *Aeromonas caviae* meningitis in adults (Table 1). In this study, we report the first case of super-refractory status epilepticus (SRSE) in a woman with *Aeromonas caviae* meningitis. We successfully managed to control her SRSE without any serious complications. Based on our case, we hope to raise clinical awareness of such diseases and provide new insights into their treatment.

**Table 1:** Characteristics of patients with aeromonas meningitis.

**Number**	**Type of infection**	**Sex**	**Age**	**Concomitant disease**	**Antibiotics**	**Outcome**	**References**
1	Aeromonas shigelloides	Female	Neonate	No	• Ampicillin • Gentamicin	Died	(4)
2	Aeromonas hydrophila	Male	35 years old	Head trauma	• Meropenem • Linezolid	Died	(5)
3	Aeromonas hydrophila	Female	2 years old	No	• Cefoperazone • Amikacin	Cured	(6)
4	Aeromonas shigelloides	Female	Neonate	No	• Rifampicin • Ampicillin	Died	(7)
5	Aeromonas hydrophila	Male	4-months-old	No	Meropenam	Cured	(8)
6	• Aeromonas veronii • biovar sobria	Male	54 years old	Alcoholic cirrhosis	Imipenem	Died	(9)
7	Aeromonas hydrophila	Male	2 years old	No	Penicillin	Died	(10)
8	Aeromonas hydrophila	Male	34 years old	Alcoholic hepatitis	• Chloramphenicol • Moxalactam • Cefotaxime	Cured	(11)
9	• Aeromonas veronii • biovar sobria	Male	66 years old	Ligation of hemorrhoids	• Cephalexin • Cefotaxime • Chloramphenicol	Cured	(12)
10	Aeromonas hydrophila	Male	39 years old	Alcoholic liver cirrhosis	NA	Died	(13)
11	Aeromonas hydrophila	Male	Neonate	No	NA	Died	(14)
12	Aeromonas hydrophila	Male	37 years old	Head trauma	• Gentamicin • Carbenicillin • Chloramphenicol	Cured	(15)
13	• Aeromonas veronii • biovar sobria	Male	40 years old	Surgery for large right temporal glomus jugulare tumor	• Ceftriaxone Cefepime • Tobramycin	Cured	(16)
14	Aeromonas hydrophila	Female	6 years old	Craniotomy with duraplasty due to Chiari malformation	• Meropenem • Trimethoprim-sulfamethoxazole	Cured	(17)
15	Aeromonas hydrophila	Male	3-months-old	No	• Ampicillin • Gentamicin • Meropenem • Cefipime	Cured	(18)
16	*Aeromonas caviae* meningitis	Female	neonate	Meningomyelocele	Cefotaxime	Cured	(3)

## Case presentation

The patient was a 47-year-old rural woman. She had had a history of hypertension for 7 years and usually managed her blood pressure with levamlodipine, which was effective. She did not have any previous neurological disorders such as stroke or epilepsy. Recently, while engaging in agricultural activities, she drank some untreated water from a natural source due to thirst and experienced abdominal pain and diarrhea, but she ignored it completely and did not seek any treatment for this discomfort.

Two days later, she experienced a sudden onset of severe headache and then collapsed and lost consciousness, accompanied by continuous seizures. Her family members immediately took her to the local hospital. Apart from a suspiciously positive Babinski sign and hyperpyrexia (39.0°C), all other physical examinations were unremarkable. Laboratory tests revealed elevated neutrophils (10.8^*^10^9^/L) and procalcitonin (1.96 ng/ml). No obvious abnormalities were found in cerebrospinal fluid (CSF) examinations or brain magnetic resonance imaging (MRI). Due to the presence of epilepsy, fever, and elevated inflammatory markers, the possibility of having an early-stage central nervous system infection could not be excluded. Considering the effective penetration of ceftriaxone through the blood–brain barrier and the inability to completely rule out viral infections, she was given ceftriaxone 3g/q8h and acyclovir 0.5g/q8h for anti-infective treatment (19). In addition, benzodiazepine (diazepam) was administered to control epilepsy (20), but no improvement was observed. She was transferred to the intensive care unit (ICU) of Jiangxi Provincial People's Hospital due to status epilepticus (SE).

**Figure 1:**
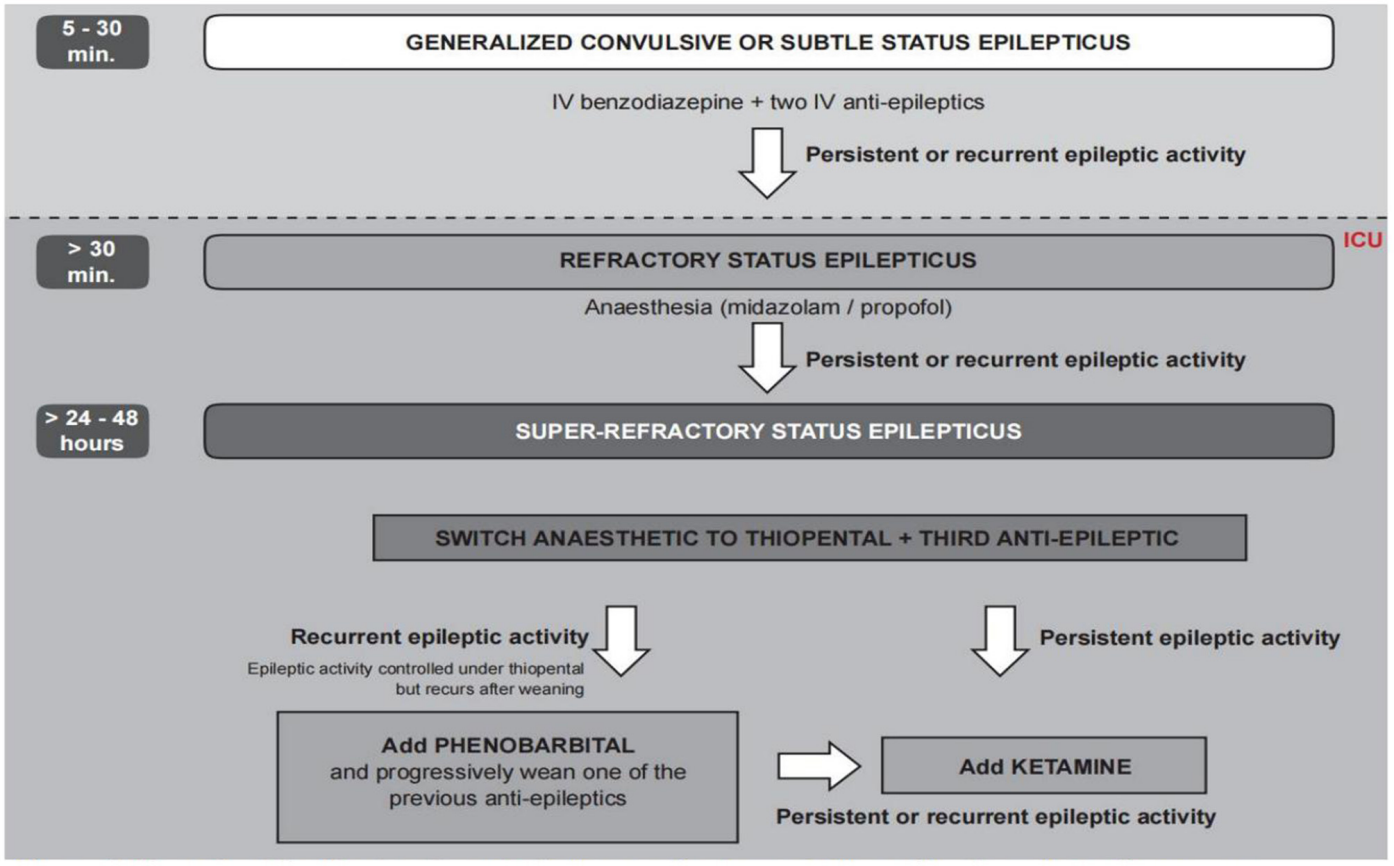
Flowchart for the treatment of super-refractory status epilepticus from consensus protocol (22) (reproduced with permission).

After admission to the ICU, we immediately gave her valproate to control the SE, but the electroencephalograph (EEG) still showed prolonged electrographic seizure activity. She was diagnosed with refractory status epilepticus (RSE) because the first- and second-line anti-epileptic medications failed (21). Therefore, we administered propofol to control her RSE based on the recommendation of consensus protocol (22) (Figure 1). Lumbar puncture for routine examination and culture of CSF was performed. We also sent a CSF sample for metagenomic next-generation sequencing (mNGS). Qualified libraries were sequenced using Darui's DA8600 platform. Its classification reference databases contained 14,330 bacteria, 814 fungi, 15,720 viral taxa, and 169 parasites. As there was no clear identification of the pathogen, the original antimicrobial treatment remained unchanged.

**Figure 2:**
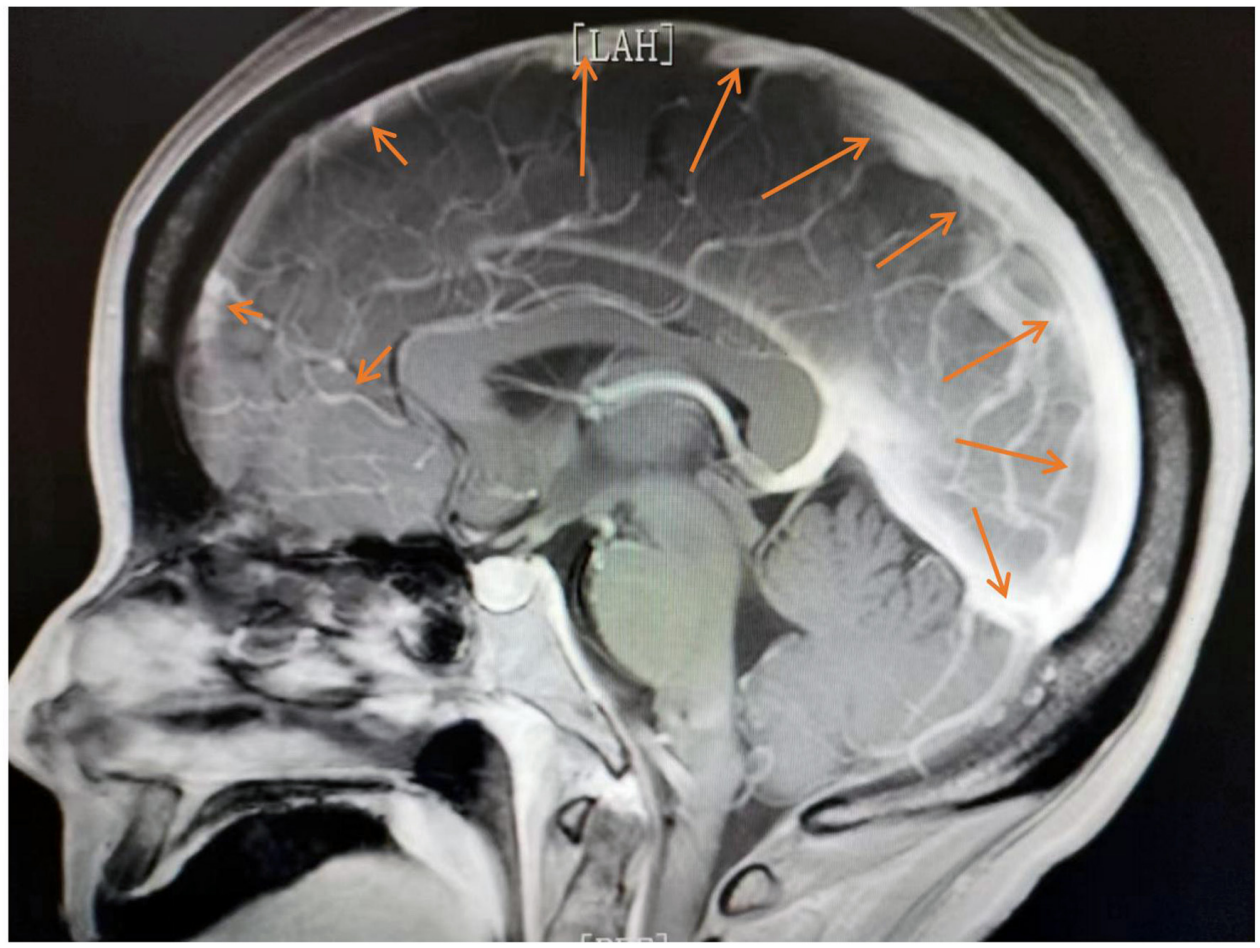
Abnormal enhancement was observed in the dura mater and pia mater.

On day 2, we performed an MRI, which showed abnormal enhancement in the dura mater and pia mater (Figure 2). No definite changes were found in the brain parenchyma. The routine CSF test indicated a positive Pandy test with a total white blood cell count of 0.06^*^10^9^/L and a protein level of 1,336 mg/L. On day 3, as she continued to exhibit recurrent epileptic activity with brief bursts of generalized spikes or generalized periodic discharges in the EEG after weaning off propofol, we considered her SRSE (23). Therefore, we added carbamazepine as a third anti-epileptic medication (22).

**Figure 3:**
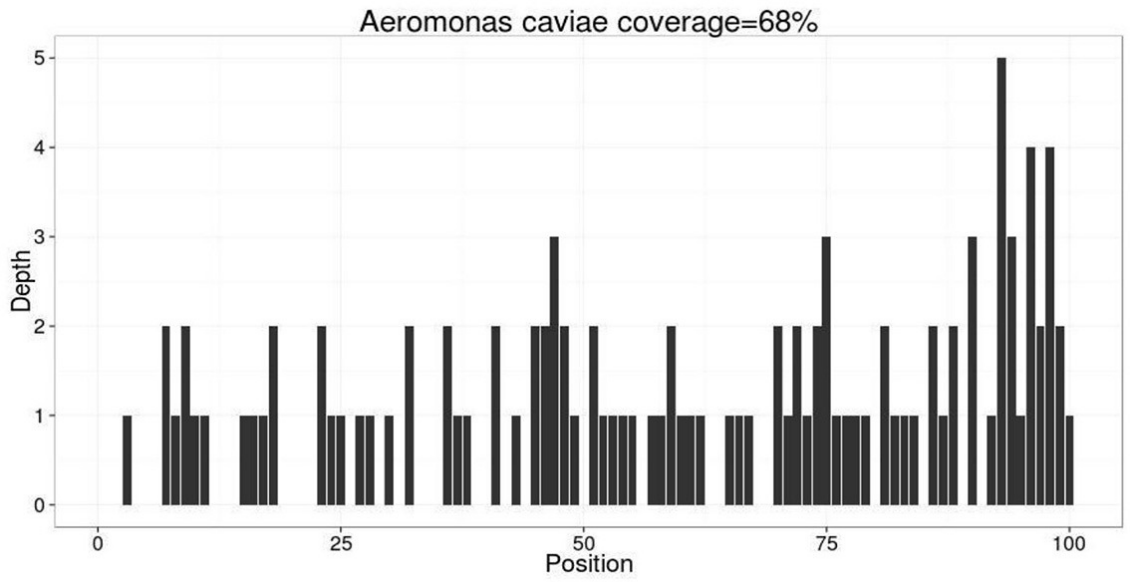
mNGS indicated 106 mapping sequence reads of *Aeromonas caviae* were identified with a coverage rate of 68%. Mapping sequence read refer to the number of sequences matched to the pathogen, which is influenced by the pathogen load in the specimen, nucleic acid extraction amount, and proportion of human sequences. A higher number indicates a higher credibility of detecting the pathogen in the specimen.

On day 4, her clinical epilepsy resolved, but there was still discontinuous epileptic electrical activity in the EEG. We introduced phenobarbital and stopped diazepam (Figure 1). Metagenomic next-generation sequencing (mNGS) indicated 106 mapping sequence reads of *Aeromonas caviae* with a 68% coverage rate, confirming an *Aeromonas caviae* infection (Figure 3). On day 5, her epileptic electrical activity completely stopped in the EEG. After discontinuing propofol, we observed that her consciousness began to improve, although her temperature and inflammatory markers remained abnormal. The CSF culture tested positive for *Aeromonas caviae*, leading to a diagnosis of *Aeromonas caviae* meningitis (19). Due to the rarity of this case, we reviewed previous literature and case reports and found reports indicating the resistance of *Aeromonas caviae* to ceftriaxone (24, 25). At the same time, a drug susceptibility test showed that *Aeromonas caviae* was highly susceptible to meropenem and resistant to ceftriaxone. Therefore, we administered meropenem 2g/q8 h to control the infection.

**Figure 4:**
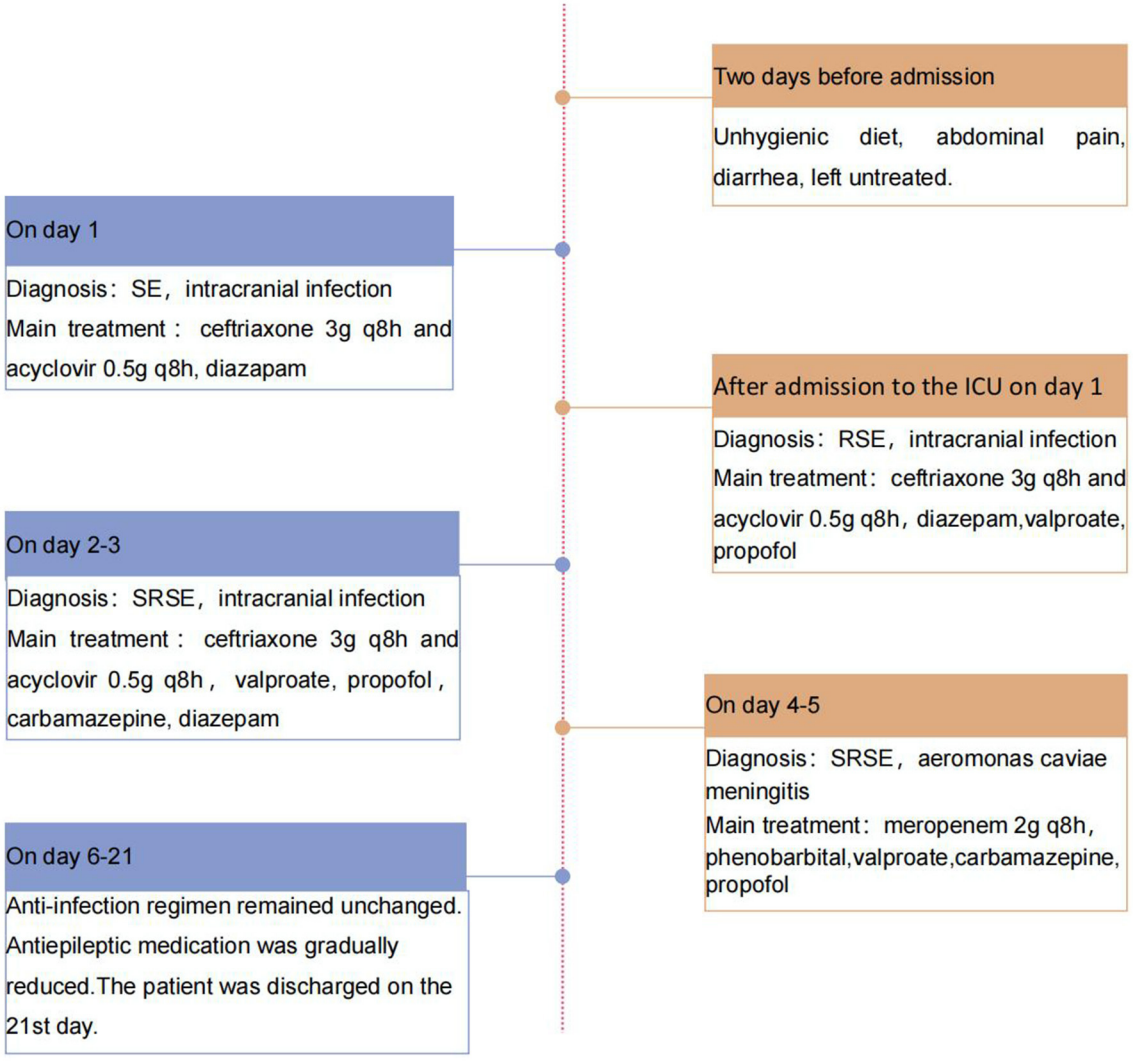
The patient's timeline of care during the hospital stay.

On day 6, her temperature and inflammatory marker levels began to decrease. On day 21, she was discharged. Figure 4 depicts her care timeline during the hospital stay. At 1-month follow-up after discharge, she exhibited no sequelae, and her brain MRI showed no significant abnormalities.

## Discussion

*Aeromonas caviae* is commonly found in aquatic environments and can contaminate water or infect aquatic animals (26). When people mistakenly drink contaminated raw water or come into direct contact with pathogens through skin wounds, it usually results in gastroenteritis (27), soft tissue infections in wounds (28), and bacteremia (29). The SRSE is an extremely rare phenomenon, as the majority of epilepsy cases are self-limiting. The incidence rate for SRSE has been reported to be 3.0/100,000 in Germany and 0.7/100,000 in Finland (30, 31). To our knowledge, this is the first reported case of SRSE in a patient with *Aeromonas caviae* meningitis. As a previously unreported type of meningitis in adults, *Aeromonas caviae* meningitis may present a significant knowledge gap in our current understanding of clinical practice, particularly when associated with SRSE.

SRSE is typically defined as status epilepticus that persists or recurs 24 h after the onset of anesthetic therapy or after its withdrawal (23). Prolonged and continuous seizure activity can lead to life-threatening complications or irreversible neurological damage. Generalized convulsive status epilepticus results in the significant release of endogenous catecholamines, which can cause arterial hypertension, potentially fatal arrhythmias, pulmonary edema requiring mechanical ventilation, renal failure, and disseminated intravascular coagulation (32). Even among survivors, severe brain injuries may occur. Lapenta et al. (33) reported a case of SRSE in a 17-year-old healthy girl who experienced mild cognitive impairment despite the resolution of seizure symptoms and recovery upon discharge from the hospital. Furthermore, the short-term mortality rate of SRSE was reported to reach 40% (34). Therefore, early recognition and timely control of SRSE are crucial for improving outcomes.

At present, there is a lack of understanding regarding *Aeromonas caviae* meningitis. Our case suggests that, without effective antibiotic intervention, this condition can lead to severe SRSE. However, there is currently no specific and unified drug treatment protocol. Once patients with *Aeromonas caviae* meningitis develop SRSE, its treatment is fraught with challenges and uncertainties. The consensus protocol (22) proposes that combining up to one anesthetic drug and three non-anesthetic anti-epileptic drugs may be helpful in managing SRSE. Combined with this protocol, we share our experience of drug use through this case, which may provide a reference for treating similar patients.

Regarding anesthesia drugs, the consensus protocol (22) suggests considering the use of propofol or midazolam when a patient presents with RSE. If treatment fails, they recommend switching to an alternative anesthesia drug while emphasizing the importance of thiopental and ketamine as substitutes. Another management review for SRSE (35) equally recommends propofol, midazolam, and pentobarbital as anesthesia drugs without considering which one is more effective. A review of SRSE (36) does not provide specific drug recommendations but reports some small-scale research where ketamine, pentobarbital, and inhalation anesthesia with isoflurane were used after failed treatment with the aforementioned drugs.

In our case, initially, we did not consider that the patient would develop SRSE. After her lack of response to diazepam, we suspected she had RSE and used propofol as an anesthetic agent for controlling epilepsy. There were two reasons why we chose propofol. First, compared to propofol, barbiturate therapy for RSE has lower success rates and a significantly longer intubation time (37). Additionally, observational studies have shown more adverse events associated with thiopental treatment for RSE (38). Second, although midazolam is considered the most commonly used anesthetic drug for RSE (39), the relapse rate after discontinuation is extremely high. A clinical retrospective study (40) found that, while midazolam controlled epilepsy in 82% of patients, 56% experienced breakthrough recurrence after stopping the drug (41). A comprehensive review of propofol concluded that, compared to midazolam, propofol has no difference in sedative effects for adult patients in the ICU. However, it does provide benefits such as low accumulation, faster recovery, and easier control of anesthesia depth (42). Nevertheless, in our case, propofol did not demonstrate strong anti-epileptic effects. Due to the specific circumstances of this case report, these findings should be interpreted with caution and verified through further research.

Non-anesthetic anti-epileptic medications play an important role in reducing anesthesia dosage and assisting in anti-epilepsy treatment. Currently, commonly used intravenous medications include phenobarbital, phenytoin, fosphenytoin, valproate, levetiracetam, and lacosamide (35). In addition, some oral anti-epileptic drugs such as carbamazepine, oxcarbazepine, and pregabalin are also utilized (36). However, the overall value supporting the clinical use of these medicines remains limited, along with low-quality evidence.

In our case, we initially administered her valproate as a second-line anti-epileptic medication (35). Maintaining respiratory and circulatory functions is vital for SRSE patients when they are experiencing frequent epilepsies. Intravenous valproate has good tolerability in terms of cardiovascular and respiratory status in patients with SRSE. Its adverse reaction rate is below 10%, with dizziness and thrombocytopenia being the most common side-effects (43). In addition, valproate is more effective than phenobarbital and phenytoin in controlling persistent epilepsy (44, 45). However, in our case, no remarkable anti-epileptic effect was observed after the use of valproate. In this case, phenobarbital stopped the epileptic electrical activity. It is recommended to add phenobarbital if recurrent epileptic activity persists after midazolam, thiopental, and propofol fail in treating SRSE (22). Pugin et al. (46) demonstrated that phenobarbital contributed to successful anesthetic weaning. The consensus protocol (2018) explicitly states that three intravenous non-anesthetic anti-epileptic medications should not be recommended for SRSE, which may be related to increased toxicity caused by excessive interactions between intravenous anti-epileptic drugs. For instance, the active component of phenytoin also increases with displacement from protein binding sites by valproate (47). Elevated drug concentrations will increase the risk of poisoning. In the context of the combined use of multiple medications, carbamazepine may be safer due to the self-inducing phenomenon where higher doses will lead to increased drug metabolism (48). Its safety was also supported by the fact that we infused carbamazepine into the stomach tube to control SRSE without adverse events such as severe liver and kidney function damage or difficulty in awakening. In addition, carbamazepine also helped control the patient's noticeable clinical episodes in our case. Thus, carbamazepine may be an alternative option to intravenous anti-epileptic drugs, but further research is still needed.

However, it must be acknowledged that our findings are based solely on one case and some clinical consensus, thereby limiting their applicability in larger populations. Although we successfully controlled SRSE, there is still a possibility of spontaneous cessation for any type of epilepsy. Therefore, our results need to be interpreted with caution. Finally, it is not yet clear whether SRSE is a common clinical manifestation or an incidental phenomenon in patients with *Aeromonas caviae* meningitis.

## Conclusion

Our report suggests that patients with *Aeromonas caviae* meningitis may experience severe SRSE. For such patients, a combination of up to one anesthetic drug and three non-anesthetic anti-epileptic drugs may be a good principle of medication selection. Our medication regimen may provide a reference for the clinical practice of *Aeromonas caviae* meningitis. Further research is needed to determine specific medications for treating SRSE in patients with *Aeromonas caviae* meningitis.

## Data availability statement

The original contributions presented in the study are included in the article/supplementary material, further inquiries can be directed to the corresponding authors.

## Ethics statement

Written informed consent was obtained from the individual(s) for the publication of any potentially identifiable images or data included in this article.

## Author contributions

YH: Writing – original draft. JL: Writing – review & editing. SW: Writing – review & editing. JC: Writing – review & editing.
